# Energy drinks and population health: consumption pattern and adverse effects among Saudi population

**DOI:** 10.1186/s12889-019-7731-z

**Published:** 2019-11-21

**Authors:** Gehad M. Subaiea, Ali F. Altebainawi, Thamir M. Alshammari

**Affiliations:** 1 Department of Pharmacology and Toxicology, College of Pharmacy, University of Hail, Hail, Kingdom of Saudi Arabia; 2 Medication Safety Research Chair, King Saud University, Riyadh, Kingdom of Saudi Arabia; 3 College of Pharmacy, University of Hail, Hail, Kingdom of Saudi Arabia; 4 Clinical Pharmacy Department, Pharmaceutical Care Services, King Khalid Hospital, Ministry of Health, Hail, Kingdom of Saudi Arabia; 5 Department of Clinical Pharmacy, College of Pharmacy, University of Hail, P.O.Box 6166, Zip code 81442 Hail City, Kingdom of Saudi Arabia; 6 Saudi Food and Drug Authority, Riyadh, Kingdom of Saudi Arabia

**Keywords:** Energy drinks (EDs), Awareness, Adverse effects, Consumption, Population, Saudi Arabia

## Abstract

**Background:**

Caffeine containing energy drinks (EDs) are heavily consumed, particularly among young adults. The number of reports of caffeine intoxication from caffeinated EDs and problems related to caffeine dependence and withdrawal is increasing. The objective was to assess the knowledge and perceived beneficial effects of EDs consumers, to assess consumption patterns and determine the adverse effects experienced by different EDs consumer groups residing in Saudi Arabia.

**Methods:**

An observational cross-sectional study with data from a randomly selected Saudi population was conducted during the period of January 15th, 2015, to April 15th, 2015, using a pre-tested 43-item questionnaire. The data were obtained and collected using interview questionnaires. Sociodemographic characteristics and data on EDs consumption patterns, the level of awareness among study subjects, and the purported benefits and reported adverse effects of EDs were collected. Frequency, percentage, and arithmetic means were calculated using Chi-square and ANOVA tests, and data with *p* < 0.05 were considered significant.

**Results:**

Of the 816 individuals invited to participate in the study, 783 participants responded and completed interviews, response rate was 96%. Consumers attributed the popularity of EDs to massive advertising media (46.7%) and their stimulating and invigorating effects (37.5%). EDs are consumed by subjects for their effects on fatigue reduction (64.6%), increased alertness and focus (75.8%), and assistance during long driving trips (75.7%). Study subjects reported suffering from adverse effects, including mainly diuresis (53.7%), palpitations (50.7%), insomnia (50.7%). Importantly, an inverse association was identified between knowledge of EDs and consumption rate, and a proportional association was identified between experienced adverse effects and consumption frequency. Lower knowledge scores were identified in daily consumers than in 1–3 times monthly consumers; higher adverse events were experienced by daily consumers than by 1–3 times monthly consumers. The majority of consumers (84.6%) recommended that authorities should regulate EDs consumption.

**Conclusions:**

Excessive EDs consumption is associated with an increased risk of experiencing several adverse events, which is commensurate with published studies. Increasing knowledge about EDs and their possible risks could decrease their consumption by the general public.

## Background

Energy drinks (EDs) are beverages that are used to boost energy, increase physical performance, increase alertness and wakefulness, and elevate mood [1]. EDs consumption is increasing, particularly among young adults and athletes [2–4]. EDs contain large doses of caffeine and other legal stimulants [1]. EDs first appeared in Asia and Europe in the 1960s; however, after the introduction of Red Bull® in Austria in 1987 and in the U.S. in 1997, the trend towards aggressive marketing of these EDs has grown tremendously. Since that time, the ED market has grown exponentially with nearly 500 new brands launched worldwide in 2006 and 200 new brands launched in the US in the 12-month period ending July 2007 [5]. The total U.S. retail market value of EDs from all sources was estimated to be $5.4 billion in 2006, and this value has shown a similar annual growth rate over this same period (47%) [5].

Caffeine containing EDs are consumed heavily among Saudis, particularly among adolescents, including females, and college students due to aggressive marketing of their overestimated ability to provide vast physical energy, which promoted their widespread use [2, 3, 6]. They are consumed to attain several effects, such as increased endurance, staying awake, fatigue relief, invigoration, memory/concentration boost and enjoyment of their taste [7]. Several EDs are available on the Saudi market, such as Red Bull®, Boozy®, Bison®, and Power Horse®. These EDs are available and easily accessible on the market, whether they are sold in small retail grocery stores or in large supermarkets. Due to this easy access to EDs and heavy marketing of these compounds in various multimedia sources, people are consuming more and more EDs [8]. Thus, the ED sector has exhibited strong worldwide growth with increased monetary rewards for companies that sell these products.

Caffeine is the key ingredient in EDs; it is also found in wide variety of beverages and some pharmaceutical products, which are commonly called psychoactive substances worldwide [9]. Caffeine has stimulating effects [10, 11] that can enhance mental and physical performance [10] and has been associated with improved alertness [12], concentration [10, 12], endurance, reaction time and mood [13, 14]. EDs typically contain high levels of caffeine, guarana, taurine, L-carnitine, vitamins, carbohydrates and sugar along with various other amino acids. According to *Gunj* et al., the typical dose of added caffeine is 7–32 mg/100 ml, giving a total dose of 35–150 mg in a 500-ml can [15]. Higher caffeine consumption is associated with increased adverse effects, including nausea, irritability and palpitations [16]. In addition, Caffeine has been demonstrated to increase the number of awakenings during sleep, sleep latency, and sleep interruptions [17]. The quantities of caffeine contained in single servings of EDs are not generally high enough to produce severe symptoms; however, at least one death has already been attributed to ED use as a 25-years old female who had pre-existing heart condition namely mitral-valve prolapse, which is present in 2.4% population, passed away after ingesting an energy drink that contained high concentrations of caffeine and guarana [18].

Two-hundred and 50 caffeine intoxication cases with an average patient age of (21) years old have been reported by the Chicago Poison Control Center, and 12 % of these cases suffered from medical complications that were attributed to EDs abuse [19]. A case study published in BMJ recently stated that in the US, approximately 23,000 emergency cases each year were due to dietary supplements [20]. Patients with pre-existing cardiac pathology or a history of seizures may be at greater risk [21–24]. Several tachyarrhythmias, including atrial and ventricular ectopic beats, atrial fibrillation, ventricular tachycardia, and ventricular fibrillation, have been described in caffeine-poisoned patients [18, 25]. Caffeine-induced seizures may occur at low doses in susceptible individuals or as a result of overdose. One case series of 4 adults with new-onset seizures questioned an association with heavy EDs use [16].

It has been documented in the literature that excessive consumption of caffeinated EDs is associated with several potentially lethal adverse effects and toxicities [21, 23, 26–29]. Caffeine containing EDs cause a rise in blood pressure (BP) due to increased systemic vascular resistance, which may worsen in severe cardiac disease, hypertension, hyperthyroidism or acute myocardial disease if precautions are not taken [30]. Additionally, methylxanthine-containing products stimulate gastric acid secretion, which should be cautiously considered in patients with peptic ulcer disease [31]. Over-the-counter (OTC) medications, such as NoDoz® and Midol®, contain between 100 and 200 mg of caffeine per tablet [32]. Some OTC drugs that are easily available for the public in the Kingdom of Saudi Arabia (KSA) contain 30–65 mg of caffeine, such as Panadol Extra® (65 mg), Adol Extra® (65 mg), Solpadine® (30 mg), and Fevadol plus® (35 mg). *McCusker* et al. reported that the U.S. Food and Drug Administration (FDA) has limited the caffeine content of sodas to 65 mg per 12 oz. (18 mg/100 ml); however, EDs are not currently subjected to the same FDA regulations [33]. According to *Nawrot,* the caffeine concentrations of various beverages are as follows: brewed coffee (56–100 mg/100 ml), instant coffee and tea (20–73 mg/100 ml), and colas (9–19 mg/100 ml) [34]. Smaller amounts can be found in chocolate (5–20 mg/100 g) and cocoa (7 mg/5 oz. cup) [32].

Several countries have demanded regulations on the labeling, distribution, and sale of EDs that contain significant amounts of caffeine. According to the European Union, Red Bull® EDs are required to have a “high caffeine content” label, and Canada requires labels indicating that Red Bull® should not be mixed with alcohol and that maximum daily consumption should not exceed two 8.3-oz cans [5]. Some countries have acted against the sale of EDs, including Red Bull®, in pharmacies, whereas France and Denmark have prohibited the sale of Red Bull® [35]. It has been suggested that warning labels on ED cans should inform the public of their high caffeine content and their potential adverse effect of raising BP [29].

In light of this, the objectives of the current study were to evaluate consumption pattern, to identify factors that influence consumption pattern, and to document the frequency of possible adverse effects of EDs among KSA population. Specifically, we aimed to obtain answers for the following research questions: How sociodemographic, levels of knowledge and perceived beneficial effects of EDs would affect their consumption pattern and intake frequencies, as well as what were the frequencies of possible adverse effects experienced by different consumer groups in the Kingdom. With that being done, we sought information about reasons for the popularity of EDs among participants, suitable replacements for EDs suggested by consumers and whether they attempted to quit consuming them. We also aimed to determine the consumption pattern of EDs with cigarette smoking as a negative health behavior in this study.

## Methods

### Study design and duration

An observational cross-sectional study of a randomly selected population in the KSA was conducted with multi-stage sampling in the period of January 15th, 2015, to April 15th, 2015, using a pre-tested 43-item questionnaire. Information from published studies was obtained to calculate the sample size for this study. The consumption of caffeine containing EDs was found to be 51% [8]. The acceptable margin of error was 5% (decided by the authors of this study), and the confidence level was 95%. Therefore, the calculated standard error was 2.55, and thus, the calculated minimum sample size was 385.

### Measures and participants

The measures used to obtain our data in our study were through utilizing a study questionnaire that was structured to consist of 43 closed-ended questions and was developed following an extensive literature review (Additional file 1). The questionnaire consisted of 5 sections with total of 43 close ended questions. The first section consisted of 8 questions to assess sociodemographic information of study participants. The second section composed of 7 questions to assess knowledge of participants about EDs. The third section consisted of 6 questions to assess patterns of consumption among study participants. The forth section consisted of 5 questions to assess the perceived positive effects believed by study participants. The last section consisted of 17 questions to assess experienced adverse events by consumers they think are associated to EDs consumption, and whether they tried to quit drinking them and experienced withdrawal symptoms. Prior to starting the study, the survey was evaluated by three faculty members with experience in the field to enhance its content and validity. Additionally, a pilot study with the survey was conducted with 15 random people to verify content clarity and accuracy. Study covered participants from several regions in KSA including Hail region (25%), Riyadh region (32%), Eastern province (15%), Jeddah region (18%) and Mecca city (10%).

The inclusion criteria required for participants in this study were the consumption of EDs at least once a month and an age between 15 and 63 years old. The age range was selected so we can subgroup consumers into those who are adolescents in high school (15–18 years), young age (19–25 years) who would be college students, and who would be in middle (26–40 years) and old age (≥41 years). People who did not meet these inclusion criteria or were unable to provide a verbal answer were excluded from this study. Ethical approval for conducting this research was reviewed and granted by the Medication and Safety Research Chair of King Saud University.

### Data collection

The survey was conducted in three phases; in the first phase, a set of high schools was randomly selected to fulfill the required number of students to participate in the survey (20% of participants). Phase two was conducted through recruiting college students (40% of participants) who were handed questionnaires that were collected on site; any questionnaire that was incomplete was not included in the study. In the third phase, general public was recruited at random malls (40% of participants) and the questionnaire was filled as an interview response. A total of 783 participants responded and completed the interview out of the 816 individuals invited to participate in the study, with a response rate of 96%.

### Statistical analysis

Descriptive statistics to accurately interpret and present the results were conducted. Chi square test was used to analyze categorical data to determine consumption patterns as related to participants’ gender, age, education level, thoughts about popularity of EDs, thoughts about possible replacements for EDs, attempts to quit EDs and smoking habits. Analysis of Variance (ANOVA) was performed to establish associations between participants’ consumption frequency and total scores of knowledge, perceived effects, and experienced side effects. In addition, ANOVA test was conducted to compare knowledge levels about EDs among different consumer subgroups, specifically those who drink EDs daily versus those who drink EDs 1–3 times monthly. Side effects experienced by daily consumers versus 1–3 times monthly were assessed as well by ANOVA. Frequencies, percentages, and arithmetic means were calculated using Chi-square and ANOVA tests on the collected data to evaluate statistical significance using SAS V.9 software.

## Results

### Sociodemographics

The majority of participants were within the 19–25- and 26–40-year-old range. The distribution of sociodemographic characteristics of the participants in this study is shown in Table 1. Most participants were male and the vast majority of participants were educated. Sleeping patterns among participants were diverse; 66.3% of participants had sleep durations ranging between 6 and 8 h, 17.3% had sleep durations of less than 5 h, and 16.4% slept more than 8 h daily. However, most study participants reported that they had sleep irregularity (Table 1).

**Table 1:** Distribution of sociodemographic characteristics of study participants and select health habits

Characteristics	Number of Subjects (n)	Percentage (%)
Gender		
Male	634	81.0
Female	149	19.0
Age Group		
15-18 Years	167	21.1
19-25 Years	289	36.6
26-40 Years	312	39.5
≥41 Years	22	2.8
Education Level		
Uneducated	11	1.4
Less than High School	52	6.6
High School Degree	239	30.4
University Student	214	27.3
B.Sc.	227	28.9
Graduate (MS-PhD)	42	5.4
Cigarette Smoking		
Yes	465	58.6
No	329	41.4
Sleeps Regularly		
Yes	311	39.5
No	476	60.5
Sleep Duration		
5 Hours or less	138	17.3
6-8 Hours	529	66.3
More than 8 Hours	131	16.4
Self-Health Awareness		
Good Health	607	76.6
Bad Health	185	23.4

### Consumption pattern

The study investigated consumption patterns among study participants (Table 2). Average consumption was divided into four groups: daily, more than once weekly, once weekly and 1–3 times monthly. More than half of the surveyed participants consumed EDs one to three times per month. Approximately, similar percentages of participants consumed EDs daily, more than once weekly, and once weekly. The results indicated that people had no specific preferred time for ED consumption, whereas quarter of the participants drank them with meals. Almost equal percentages of study population drank them in the morning and at night. Of eight famous brands of EDs, Black® and Red Bull® were the most brands consumed by study participants mostly for their good taste (Table 2).

**Table 2:** Distribution of energy drink consumption pattern among study participants

Characteristics	Number of Subjects (n)	Percentage (%)
Average Consumption of Energy Drinks		
Daily	144	18.2
More than Once Weekly	150	18.9
Once Weekly	97	12.2
1-3 Times Monthly	402	50.7
Energy Drinks Preferred Consumption Time		
In the Morning	111	14.4
With Meals	207	26.8
At Night	154	19.9
Anytime	301	39.3
Activities Related to Energy Drinks Consumption		
Meetings and Celebrations	257	32.4
Study Exams	275	34.7
Others	261	32.9
Preferred Energy Drink Brand		
Bison	135	17
Red Bull	192	24.2
Power Horse	108	13.6
Bugzy	25	3.2
Code Red	229	28.9
Black	1	0.1
Smart Cola	7	0.9
Vitamin C	96	12.1
Reason for Selection of Preferred Brand		
Taste	513	64.7
Strong Effect	115	14.5
Price	88	11.1
Other	77	9.7

### Adverse effects experienced

We also aimed to identify the prevalence of adverse effects associated with ED intake. The study subjects reported suffering from various adverse events as shown in Table 3. Study subjects mainly reported suffering from diuresis, palpitations, insomnia, tooth decay, lack of rest and confusion. Muscle weakness and rigidity, nervousness, nausea/abdominal pain, tiredness and chronic fatigue, headache, tremors, chest pain, and constipation, were reported less frequently.

**Table 3:** Adverse effect experienced by energy drink consumers who participated in the study

Characteristics	Number of Subjects (n)	Percentage (%)
Side Effects Experienced		
Chest Pain	218	27.8
Palpitations	397	50.7
Insomnia	397	50.7
Headache	226	29
Constipation	193	24.7
Diuresis	418	53.4
Chronic Fatigue	237	30.3
Tooth Decay	376	48
Muscle Fatigue	262	33.5
Lack of Rest	351	44.8
Tremors	225	28.7
Confusion	342	43.7
Nausea/Abdominal Pain	241	30.8
Nervousness	253	32.3

### Sociodemographics and consumption pattern

We revealed important findings by examining the participants’ sociodemographic data and average consumption patterns. Even though females were found to consume EDs less frequently than males (19.2% daily in males vs. 14.8% daily in females, 49.2% 1–3 times monthly in males vs. 57.7% 1–3 times monthly in females), the results did not reach statistical significance (X^2^ = 5.49, *p* = 0.136 - Table 4). However, an association between age and consumption patterns exists as we found that younger participants consumed EDs more frequently than older groups and that the percentage of least frequent consumers (i.e., the 1–3 times monthly group) increased with age. As shown in Table 4, 25.7% of the 15–18 year-old participants consumed EDs daily compared to 16.3% in the 19–25- and 26–40-year-old consumers and 13.6% in participants 41 years of age or older (*p* < 0.001). The percentage of participants with the lowest ED consumption pattern (1–3 times monthly) increased significantly from 39.5% in 15–18-year-old group to 50.5% in 19–25-year-old group to 55.4% in the 26–40-year-old group to 68.2% in the ≥41 year-old group (X^2^ = 26.13, *p* < 0.001 – Table 4). In addition, educational level significantly altered average consumption patterns (X^2^ = 46.76, *p* < 0.001 – Table 4), as uneducated and participants with less than a high school diploma had the lowest percentages among the 1–3 times monthly group (27.3 and 38.5%, respectively, vs. 50% + in higher educational level groups).

**Table 4:** Analysis of different average consumption groups of study participants and their gender, age and education level

	Daily		>1 Weekly		Once Weekly		1-3 Times Monthly		Total		Chi (X^2^)
	(n)	(%)	(n)	(%)	(n)	(%)	(n)	(%)	(n)	(%)	*p* Value
Gender											5.49
Male	122	19.2	126	19.9	74	11.7	312	49.2	634	81.0	0.136
Female	22	14.8	21	13.1	20	13.4	94	57.7	149	19.0	
Age Group											26.13
15-18 Years	43	25.7	27	16.2	31	18.6	66	39.5	167	21.1	<0.001*
19-25 Years	47	16.3	58	20.1	38	13.1	146	50.5	289	36.6	
26-40 Years	51	16.3	61	19.6	27	8.7	173	55.4	312	39.5	
≥41 Years	3	13.6	4	18.2	0	0.0	15	68.2	22	2.8	
Education Level											46.75
Uneducated	4	36.4	3	27.3	1	9.1	3	27.3	11	1.4	<0.001*
Less than High School	6	11.5	8	15.4	18	34.6	20	38.5	52	6.6	
High School Degree	52	21.7	42	17.9	25	10.4	120	50.0	239	30.4	
University Student	22	10.3	45	21.0	30	14.0	117	57.7	214	27.3	
B.Sc.	50	22.1	41	17.7	18	8.0	118	52.2	227	28.9	
Graduate (MS-PhD)	9	21.4	7	16.7	3	7.1	23	54.8	42	5.4	

### Popularity of EDs and consumption pattern

Additionally, participants were asked for their opinion on the popularity of EDs in the community (Table 5). When analyzing the four different average consumption patterns of the study participants and their thoughts on why EDs were popular, a significant difference in thoughts was revealed as 45.8% of daily consumers attributed ED popularity to their invigorating effect compared to 33.7% of the 1–3 times per month consumers (Table 5). However, the majority of 1–3 per month consumers (53.1%) attributed the popularity of EDs to intense media commercials (X^2^ = 19.66, *p* = 0.0032, Table 4). Different thoughts about suitable replacements for EDs were also identified. Significantly, the 1–3 times monthly consumer group was the least likely to consider coffee as a suitable replacement for EDs (36.6%) compared to the daily and once weekly users (46.5 and 45.8%, respectively – X^2^ = 37.1, *p* < 0.001). However, the 1–3 times monthly consumer group was the most likely to attempt quitting ED consumption compared to the other groups (64% vs. 57.7% for daily, 59.5% for > 1 weekly and 52.1% for once weekly consumers – X^2^ = 17.55, *p* = 0.0005, Table 5).

**Table 5:** Analysis of different average consumption groups of study participants and their thoughts about the reasons for energy drink popularity, suitable energy drink replacements, and whether they attempted to quit drinking energy drinks

	Daily		>1 Weekly		Once Weekly		1-3 Times Monthly		Total		Chi (X^2^)
	(n)	(%)	(n)	(%)	(n)	(%)	(n)	(%)	(n)	(%)	*p* Value
Popularity of Energy Drinks Is Due to:											19.66
Invigorating Effect	66	45.8	57	38.0	39	40.2	132	33.7	294	37.5	0.0032*
Popularity among Athletes	20	13.9	30	20.0	21	21.6	52	13.3	123	15.7	
Intense Media Commercials	58	40.3	63	42.0	37	38.1	208	53.1	366	46.7	
Suitable Replacement for Energy Drinks											37.10
Coffee	65	45.8	62	41.3	45	46.4	147	36.6	320	40.4	<0.001*
Tea	31	21.5	45	30.0	21	21.6	62	15.4	159	20.1	
Natural Herbs	36	25.0	27	18.0	15	15.5	110	27.4	188	23.7	
Others	11	7.6	16	10.6	16	16.5	83	20.6	126	15.9	
Attempts to Quit Energy Drinks											17.55 0.0005*
Yes	79	57.7	88	59.5	50	52.1	281	70.8	498	64.0	
No	58	42.3	60	40.5	46	47.9	116	29.2	280	36.0	

### Knowledge about EDs and consumption pattern

**Fig. 1:**
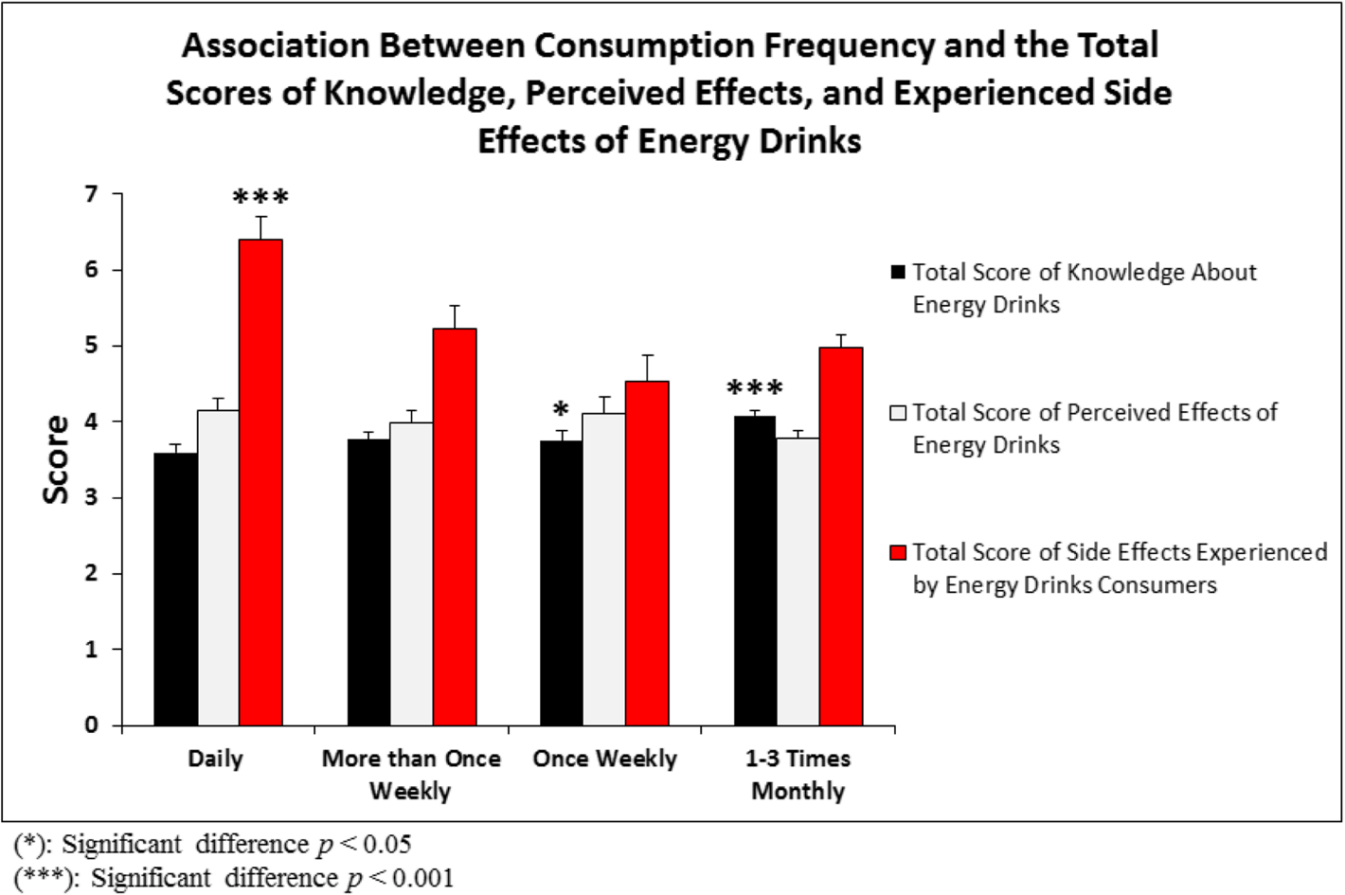
Association between Consumption Frequency and the Total Scores of Knowledge, Perceived Effects, and Experienced Side Effects of Energy Drinks

Informative results were obtained by analyzing the four consumption patterns of study participants. Significant difference in knowledge among the different consumer groups was identified (*p* = 0.0002) as the total knowledge score (involving knowledge about EDs’ effects on BP, sugar level, heart rate (HR), possible adverse effects, recommendation to others and necessity of regulation by the Saudi Food and Drug Authority (SFDA) was significantly higher in the group that consumed EDs the least than in the group that consumed EDs daily or once weekly as analyzed by ANOVA (*p* < 0.001 and *p* < 0.05, respectively – Fig. 1). Even though the total score of perceived beneficial effects about EDs (involving feeling mood elevation and increase in vitality/energy, helping with athletic/academic performance, feeling an increase in focus/memory and helping with long-distance driving) was the highest among the daily consumers compared to the rest of the consumption pattern groups (4.14 in daily consumers vs. 3.98 in more than once weekly, 4.1 in the weekly consumer group and 3.79 in the 1–3 times monthly group), this difference did not reach statistical significance (*p* = 0.22). However, the total score of adverse effects attributed to the consumption of EDs (involving chest pain, palpitation, insomnia, headache, constipation, diuresis, chronic fatigue, muscle fatigue, tremors, teeth decay, lack of rest, nervousness, nausea/abdominal pain, and confusion) was significantly different (*p* < 0.001) among the four consuming groups as daily users experienced significantly more adverse effects than the other three consuming groups (daily vs. > 1 weekly, *p* < 0.05; daily vs. weekly, *p* < 0.001; and daily vs. 1–3 monthly, *p* < 0.001 – Fig. 3).

**Fig. 2:**
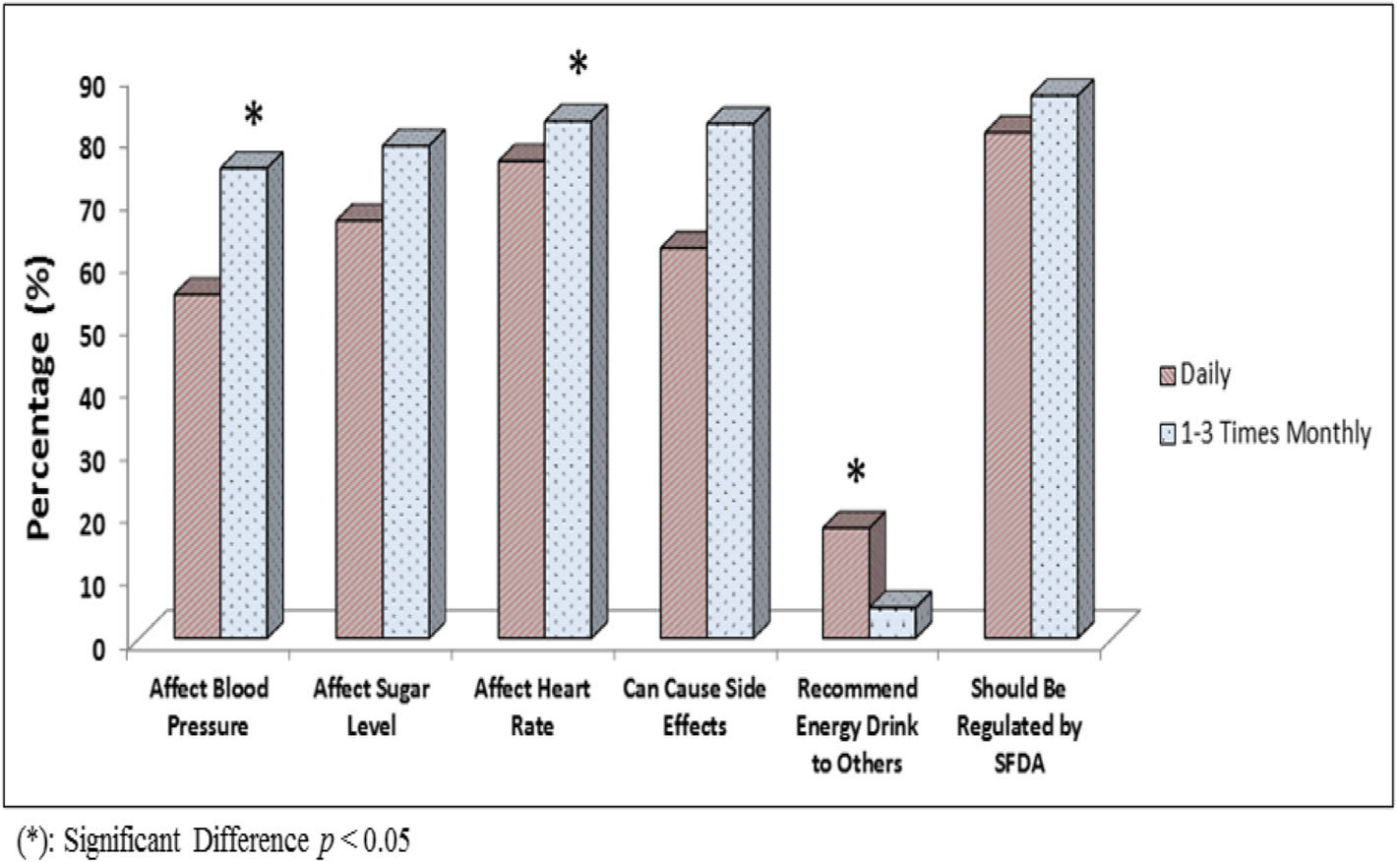
Comparison of the Knowledge about Energy Drinks between Daily and 1–3 Times per month Consumers of Energy Drinks

We compared the group that consumed EDs the most versus the group that consumed EDs the least to closely examine data regarding ED knowledge as shown in Fig. 2. Consistently higher knowledge regarding the deleterious effects of EDs was detected in the group that consumed EDs the least, which was significant for knowledge about EDs’ effects on BP and HR as analyzed by Chi-square (X^2^ = 26.18, *p* < 0.001; X^2^ = 12.15, *p* = 0.007, respectively). As a result of this difference, a significantly higher percentage of participants recommended EDs to others in the group who had the lowest knowledge score, the daily consumers (17.6% of daily consumers vs. 10.9% of 1–3 times per month consumers; X^2^ = 31.6, *p* < 0.001). The majority of both subgroups recommended SFDA initiate actions to regulate EDs consumption without statistical difference between both subgroups. In fact, most of the study participants (84.6%) recommended that SFDA should regulate EDs consumption in KSA.

### Consumption pattern and experienced adverse effects

**Fig. 3:**
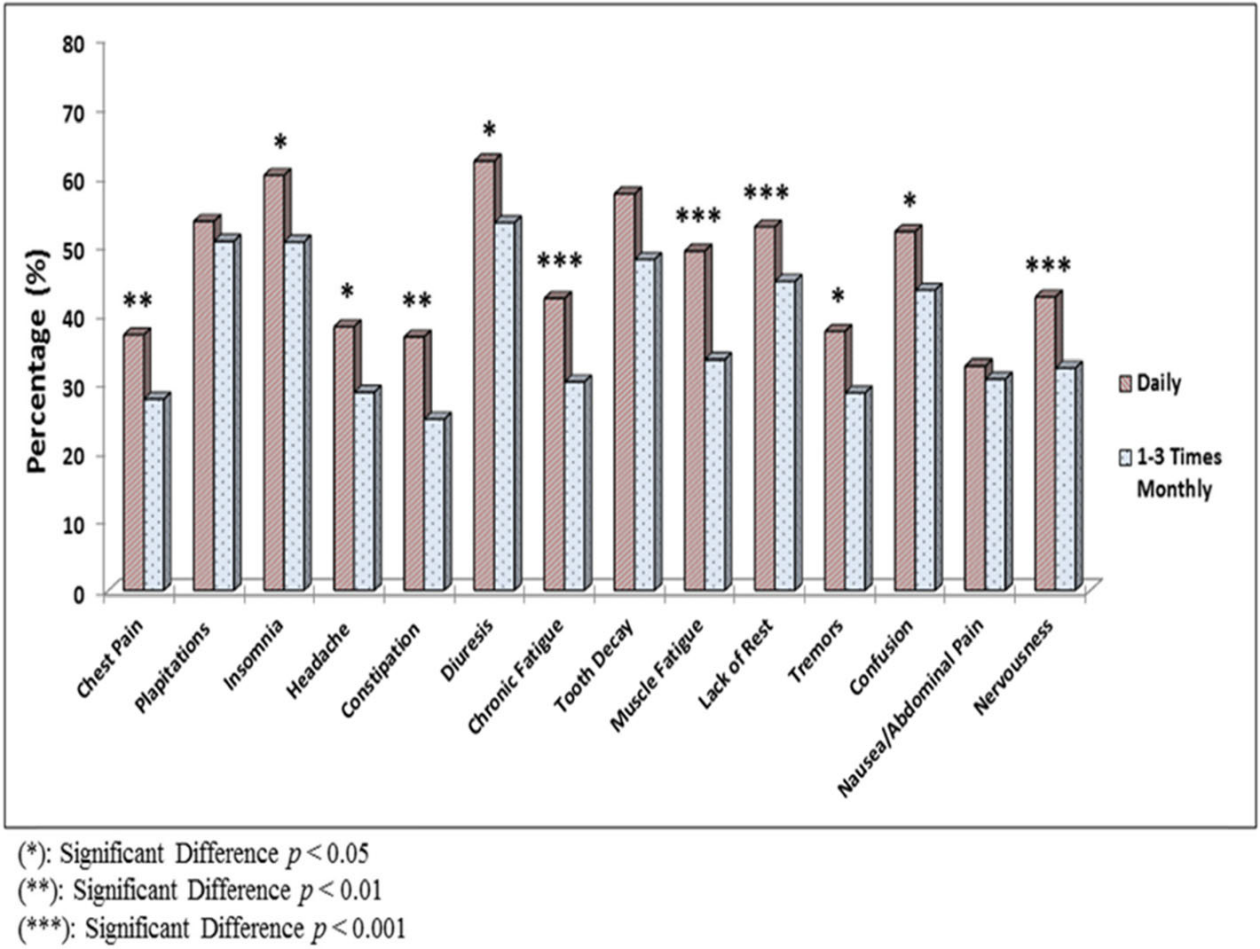
Analysis of the side effects experienced by daily energy drinks consumers versus 1–3 monthly consumers

**Fig. 4:**
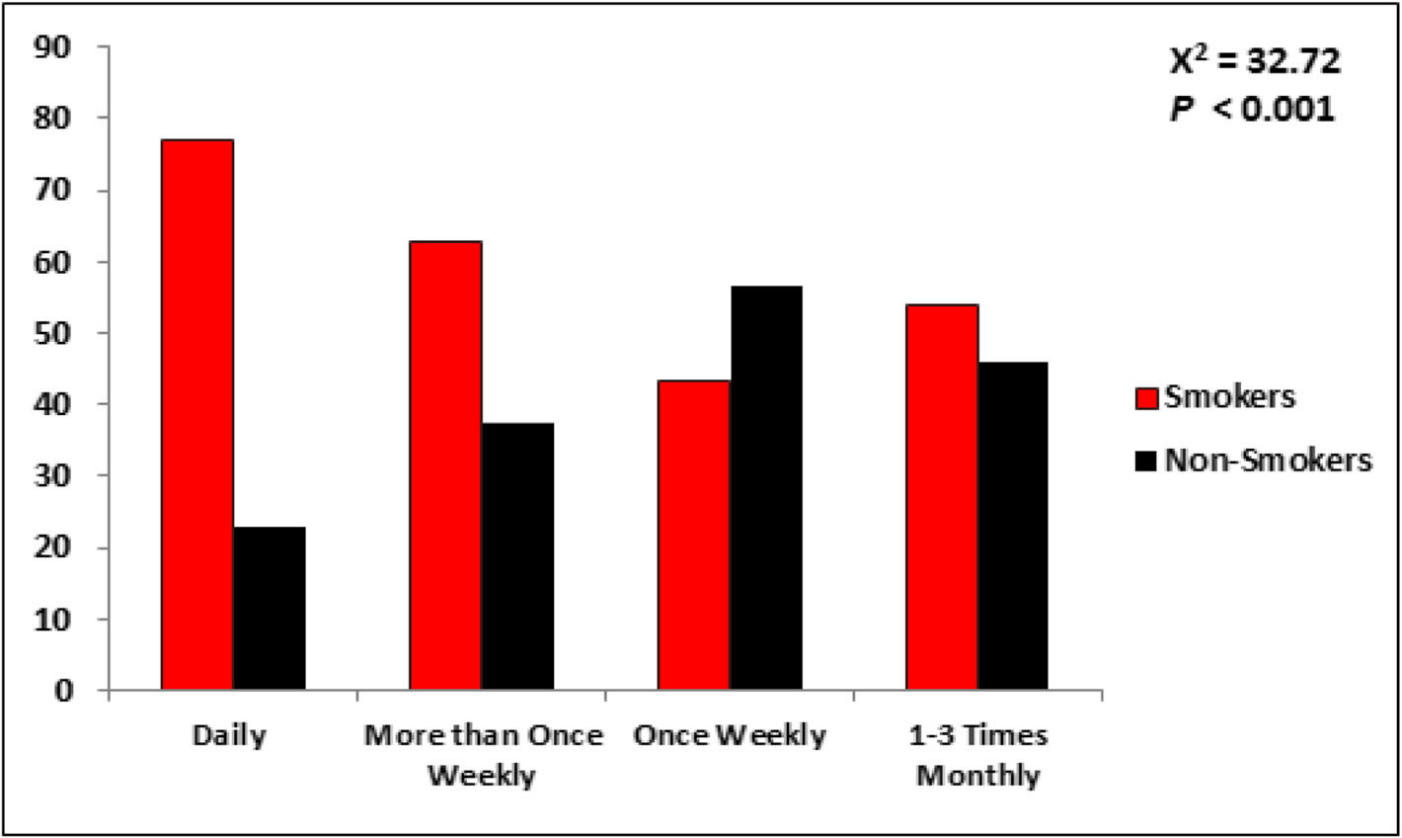
Pattern of consumption of energy drinks between smokers and non-smokers

Significant results were obtained by analyzing data on the adverse effects experienced by EDs consumers. All adverse effects evaluated by the questionnaire, which included chest pain, palpitations, insomnia, headache, constipation, diuresis, chronic fatigue, tooth decay, muscle fatigue, lack of rest, tremors, confusion, nausea/abdominal pain, and nervousness, were experienced at a higher a rate in daily consumers than in 1–3 times monthly consumers (Fig. 3). An important observation in this study is that there was a significant association between the frequency of ED consumption and smoking. As shown in Fig. 4, a higher percentage of the study participants who consumed EDs daily or more than once weekly were smokers than in the rest of the groups (X^2^ = 37.72, *p* < 0.001).

## Discussion

It has been documented in the literature that excessive consumption of caffeinated EDs is associated with several potentially lethal adverse effects and toxicities [21, 23, 26–29]. While several studies were conducted to evaluate the prevalence of EDs consumption in the KSA [2, 3, 36, 37], to the best of our knowledge, this study is the first to elaborate factors that influence consumption patterns and rates among EDs drinkers and assess the adverse effects experienced by consumers.

Here, we report that gender did not affect average consumption patterns; even though the percentage of males consuming EDs daily or more than once daily was slightly higher than that of females, this difference was not statistically significant. A published study reported that the frequency of intake among both genders was affected by age [38]; however, that research group examined only participants between 14 and 19 years old, whereas we studied consumption patterns in a much wider age range (15–63 years old). It is important to mention that previous studies in the KSA have reported that young adolescent males drink more EDs than females [3, 36, 38]. After careful examination of the collected data, we found that the vast majority of consumers were 40 years old or younger (97%); they were rarely older than 40 years old. Consumption patterns were found to be altered by age as we found that younger study participants significantly consumed EDs more frequently than older drinkers; this pattern was consistent among all age groups, and this finding was similar to those of several previous studies [38, 39].

Educational level was found to determine the intake habits of EDs. We found that consumers who did not hold a high school diploma or were uneducated drank EDs more frequently than consumers with a high school diploma or above. However, almost 90% of study participants were equally distributed between high school graduates, undergraduate students or B.Sc. holders and above, and more than half of these EDs consumers drank EDs one to 3 three times per month. However, it is crucial to mention that the prevalence of EDs consumption in schools and colleges in the KSA and in many countries worldwide is very high, reaching nearly 50% and higher [2, 3, 36, 37, 40]. According to Sandra D. Reid et al., the usage of EDs by university students is a primary concern, as these students are the ideal target of companies promoting EDs [41]. Previous reports have shown that EDs usage is prevalent among undergraduate university students in the US and that 39–80% of these students used EDs at least once in the past [42]. According to Kopacz et al., 49% of university students in Poland used EDs during the academic year, whereas ED consumption significantly increased during examination times [43]. Hence, based on this information, it is crucial to create awareness campaigns about EDs for high school and university students.

Study participants attributed the popularity of EDs to intense advertisements in different media and their perceived invigorating effects. Other studies conducted in the KSA have reported similar results [3, 37]. EDs are promoted for their effects, including boosting energy, wakefulness, and alertness and improving physical and mental performance [41]. We did not find a significant difference in the purported beneficial effects of EDs among different consumption groups. Consumers in the KSA drank EDs to attain vitality and energy, alertness, mood elevation, focus and memory and to help them on long-distance driving trips around the vast Kingdom. It has been documented that moderate intake of EDs enhances physical and cognitive functions. In fact, several studies have proven that the active ingredients in Red Bull®, caffeine and taurine, improve physical endurance and psychomotor performance [10, 44]. Study participants expressed different thoughts about suitable replacements for EDs, and they mostly stated that coffee, tea and natural herbs were their choice of beverage instead of EDs.

Importantly, we deduced from our data that lack of knowledge is associated with heavier EDs intake. Total scores of knowledge about possible adverse effects of EDs was significantly less in daily EDs consumers than in the least frequent consumer group. Specifically, significant differences between daily drinkers and 1–3 times monthly drinkers in knowledge about harmful effects on the cardiovascular system were found. Fewer daily consumers knew about the possible rise in BP and HR by EDs than those in the least frequent EDs consumer group. Furthermore, more daily drinkers recommended EDs to others. In fact, it was astonishing to discover that other studies in the KSA have indicated a lack of knowledge of even the active ingredients in EDs. Musaiger et al. found that 47% of males and 52.3% of females who consumed EDs in the KSA were not aware of ED ingredients [3]. Aluqmany et al. reported that 69.3% of high school female students in Almadinah Almunawwarah, KSA, did not know about the ingredients in EDs [37]. Another study conducted in the United Arab Emirates (UAE) reported that 95% of university students were not aware of the high caffeine levels in EDs [45]. As the EDs market is expanding rapidly both regionally and globally, consumers should also take precautions and be familiar with the ingredients contained in EDs, such as vitamins; other EDs ingredients, such as niacin, exceed the recommended daily doses, which may lead to potential adverse effects and toxicity due to accumulation [46]. Together with our findings, these observations are disturbing, and promoting knowledge about EDs content and their possible adverse effects would probably decrease their intake frequency and hence reduce the health hazards associated with excessive intake.

A significant achievement of the current study was providing further evidence of possible health risks and adverse events related to EDs intake at different consumption levels. We report several adverse effects experienced by the study participants, and their percentages. More importantly, total score analysis of experienced adverse effects, indicates a significant difference between the daily EDs consumer group versus the 1–3 times monthly consumer group as the daily users reported experiencing more adverse effects that they attributed to their intake of EDs. Ten of these adverse events, namely, chest pain, insomnia, headache, constipation, diuresis, chronic fatigue, muscle fatigue, lack of rest, tremors, confusion and nervousness were significantly experienced more frequently in daily consumers than in the lowest EDs consuming group. While some studies estimated the frequencies of experienced adverse events among EDs drinkers in the KSA [2, 47], our study is likely the first in the KSA to provide evidence of the increased occurrence of adverse effects concomitant with increased EDs intake.

We also aimed to determine the consumption pattern of EDs with cigarette smoking as a negative health behavior in this study. The cigarette smoking rate in the KSA is high, reaching up to 37.6% in males and 6% in females [48]. Approximately 76.6% of the participants in our study reported being healthy despite the fact that significantly most EDs consumers in this study were cigarette smokers (58.6%), Moreover, we found that tobacco smoking determined consumption patterns as the majority of daily EDs consumers were smokers (77%) compared to 54% tobacco smoking among the 1–3 times monthly EDs consumers. Heavier EDs intake by tobacco smokers than non-smokers might be explained by the fact that tobacco smoking induces CYT P450 1A2, which enhances the metabolism of caffeine [49], hence requiring higher caffeinated ED intake. These findings are consistent with the findings of other studies that aimed to determine EDs and associated factors worldwide [40, 50]. Another negative behavior that can be associated with EDs consumption is alcohol intake. However, alcohol drinking is prohibited by Islam and banned in the KSA; thus, it was not feasible to study this association even though some people might drink alcohol illegally. It is worth mentioning that compelling evidence has been established regarding the consequences of mixing alcohol and EDs in a single alcoholic beverage (alcohol mixed EDs – AmEDs), which can lead to increased risks of adverse effects [51, 52]. It is important to mention that recent studies have shown that caffeinated EDs cause addiction and dependence [5]. When caffeine containing EDs are taken in high amounts, they induce arousal by caffeine’s effect on the pleasure area of brain, which is similar to the effects of tobacco, alcohol, and other drugs that should be treated with caution to avoid abuse [53].

We believe our study has several points of strength, and potential limitations. To our knowledge, it is the first study that assessed the patterns of consumption as well as the adverse effects of energy drinks on different age groups of Saudi population. The study was a national one as the participants were from all over the KSA. The study questionnaire consisted of 43 items to address most of the research questions being investigated, with a very good response rate (96%). However, as the nature of the study was an observational one, it was not possible to assess causality relationship especially when assessing the adverse reaction. The majority of participants were males and only 19% were females. When reporting adverse events, the difference between groups may be, at least in part, associated with other health conditions rather than solely being related to EDs consumption.

## Conclusions

In conclusion, EDs intake is associated with many deleterious health effects that are proportional to intake level. Lack of knowledge is associated with heavier EDs intake. Young adolescents and high school and university students were found to consume EDs more frequently and at an increased risk of health effects. It is imperative, based on our findings, that regulatory authorities, including the SFDA, enact stricter regulations to control EDs marketing in the KSA. The level of awareness of possible negative health effects due to excessive EDs consumption must rise by assistance of different governmental entities, specifically, ministry of health and ministry of education.

## Supplementary information

**Additional file 1.** Study Questionnaire: The English version of the questionnaire that was used to obtain the data from study participants.

## Data Availability

The datasets used and/or analyzed during the current study are available from the corresponding author upon reasonable request.

## Abbreviations

BP: Blood Pressure
ED: Energy Drink
FDA: U.S. Food and Drug Administration
HR: Heart Rate
KSA: Kingdom of Saudi Arabia
OTC: Over-the-counter
SAS: Statistical Analysis Software
SFDA: Saudi Food and Drug Authority
U.S: United State

## References

[1] Ishak WW, Ugochukwu C, Bagot K, Khalili D, Zaky C (2012). Energy drinks: psychological effects and impact on well-being and quality of life-a literature review. Innov Clin Neurosci.

[2] Rahamathulla MP (2017). Prevalence, side effects and awareness about energy drinks among the female university students in Saudi Arabia. Pak J Med Sci.

[3] Musaiger A, Zagzoog N (2013). Knowledge, attitudes and practices toward energy drinks among adolescents in Saudi Arabia. Global J Health Sci.

[4] Nowak Dariusz, Jasionowski Artur (2016). Analysis of Consumption of Energy Drinks by a Group of Adolescent Athletes. International Journal of Environmental Research and Public Health.

[5] Reissig CJ, Strain EC, Griffiths RR (2009). Caffeinated energy drinks--a growing problem. Drug Alcohol Depend.

[6] Alsubaie ASR (2017). Consumption and correlates of sweet foods, carbonated beverages, and energy drinks among primary school children in Saudi Arabia. Saudi Med J.

[7] Alsunni A, Badar A. Energy drinks consumption pattern, perceived benefits and associated adverse effects amongst students of University of Dammam, Saudi Arabia. Ayub Med Coll Abbottabad. 2011;23(3):3–9. 23272423

[8] Malinauskas BM, Aeby VG, Overton RF, Carpenter-Aeby T, Barber-Heidal K (2007). A survey of energy drink consumption patterns among college students. Nutr J.

[9] Gilbert RM (1984). Caffeine consumption. Prog Clin Biol Res.

[10] Alford C, Cox H, Wescott R (2001). The effects of red bull energy drink on human performance and mood. Amino Acids.

[11] Heckman MA, Sherry K, De Mejia EG (2010). Energy drinks: an assessment of their market size, consumer demographics, ingredient profile, functionality, and regulations in the United States. Compr Rev Food Sci Food Saf.

[12] Warburton David, Bersellini Elisabetta, Sweeney Eve (2001). An evaluation of a caffeinated taurine drink on mood, memory and information processing in healthy volunteers without caffeine abstinence. Psychopharmacology.

[13] Miller Kathleen E. (2008). Wired: Energy Drinks, Jock Identity, Masculine Norms, and Risk Taking. Journal of American College Health.

[14] Seidl R., Peyrl A., Nicham R., Hauser E. (2000). A taurine and caffeine-containing drink stimulates cognitive performance and well-being. Amino Acids.

[15] Gunja N, Brown JA (2012). Energy drinks: health risks and toxicity. Med J Aust.

[16] Iyadurai SJ, Chung SS (2007). New-onset seizures in adults: possible association with consumption of popular energy drinks. Epilepsy Behav.

[17] Pollak CP, Bright D (2003). Caffeine consumption and weekly sleep patterns in US seventh-, eighth-, and ninth-graders. Pediatrics.

[18] Cannon ME, Cooke CT, McCarthy JS (2001). Caffeine-induced cardiac arrhythmia: an unrecognised danger of healthfood products. Med J Aust.

[19] McCarthy DM, Mycyk MB, DesLauriers CA (2008). Hospitalization for caffeine abuse is associated with abuse of other pharmaceutical products. Am J Emerg Med.

[20] Geller AI, Shehab N, Weidle NJ, Lovegrove MC, Wolpert BJ, Timbo BB, Mozersky RP, Budnitz DS (2015). Emergency department visits for adverse events related to dietary supplements. N Engl J Med.

[21] Gray B, Das KJ, Semsarian C (2012). Consumption of energy drinks: a new provocation test for primary arrhythmogenic diseases?. Int J Cardiol.

[22] Alsunni A, Majeed F, Yar T, AlRahim A, Alhawaj AF, Alzaki M (2015). Effects of energy drink consumption on corrected QT interval and heart rate variability in young obese Saudi male university students. Ann Saudi Med.

[23] Calabro RS, Italiano D, Gervasi G, Bramanti P (2012). Single tonic-clonic seizure after energy drink abuse. Epilepsy Behav.

[24] Dikici Suber, Saritas Ayhan, Besir Fahri Halit, Tasci Ahmet Hakan, Kandis Hayati (2013). Do energy drinks cause epileptic seizure and ischemic stroke?. The American Journal of Emergency Medicine.

[25] Shum S, Seale C, Hathaway D, Chucovich V, Beard D (1997). Acute caffeine ingestion fatalities: management issues. Vet Hum Toxicol.

[26] Kozik TM, Shah S, Bhattacharyya M, Franklin TT, Connolly TF, Chien W, Charos GS, Pelter MM (2016). Cardiovascular responses to energy drinks in a healthy population: the C-energy study. Am J Emerg Med.

[27] Venkatraman A, Khawaja A, Shapshak AH (2017). Hemorrhagic stroke after consumption of an energy drink. Am J Emerg Med.

[28] Dikici Suber, Saritas Ayhan, Besir Fahri Halit, Tasci Ahmet Hakan, Kandis Hayati (2013). Do energy drinks cause epileptic seizure and ischemic stroke?. The American Journal of Emergency Medicine.

[29] Usman A, Jawaid A (2012). Hypertension in a young boy: an energy drink effect. BMC Res Notes.

[30] Grasser EK, Miles-Chan JL, Charriere N, Loonam CR, Dulloo AG, Montani JP (2016). Energy drinks and their impact on the cardiovascular system: potential mechanisms. Adv Nutr.

[31] Stoller JL (1985). Oesophageal ulceration and theophylline. Lancet.

[32] Benowitz NL (1990). Clinical pharmacology of caffeine. Annu Rev Med.

[33] McCusker RR, Goldberger BA, Cone EJ (2006). Caffeine content of energy drinks, carbonated sodas, and other beverages. J Anal Toxicol.

[34] Nawrot P, Jordan S, Eastwood J, Rotstein J, Hugenholtz A, Feeley M (2003). Effects of caffeine on human health. Food Addit Contam.

[35] Higgins JP, Tuttle TD, Higgins CL (2010). Energy beverages: content and safety. Mayo Clin Proc.

[36] Faris M (2014). Patterns of caffeinated energy drinks consumption among adolescents and adults in hail, Saudi Arabia. Food Nutr Sci.

[37] Aluqmany R, Mansoor R, Saad U, Abdullah R, Aa A (2013). Consumption of energy drinks among female secondary school students, Almadinah Almunawwarah, Kingdom of Saudi Arabia, 2011. J Taibah Univ Med Sci.

[38] Al-Hazzaa HM, Abahussain NA, Al-Sobayel HI, Qahwaji DM, Musaiger AO (2011). Physical activity, sedentary behaviors and dietary habits among Saudi adolescents relative to age, gender and region. Int J Behav Nutr Phys Act.

[39] Bunting H, Baggett A, Grigor J (2013). Adolescent and young adult perceptions of caffeinated energy drinks. A qualitative approach. Appetite.

[40] Attila S, Cakir B (2011). Energy-drink consumption in college students and associated factors. Nutrition.

[41] Reid SD, Ramsarran J, Brathwaite R, Lyman S, Baker A, Cornish DC, Ganga S, Mohammed Z, Sookdeo AT, Thapelo CK (2015). Energy drink usage among university students in a Caribbean country: patterns of use and adverse effects. J Epidemiol Glob Health.

[42] Hoyte CO, Albert D, Heard KJ (2013). The use of energy drinks, dietary supplements, and prescription medications by United States college students to enhance athletic performance. J Community Health.

[43] Kopacz A, Wawrzyniak A, Hamulka J, Gornicka M (2013). Evaluation of energy drink intake in selected student groups. Rocz Panstw Zakl Hig.

[44] Warburton DM, Bersellini E, Sweeney E (2001). An evaluation of a caffeinated taurine drink on mood, memory and information processing in healthy volunteers without caffeine abstinence. Psychopharmacology.

[45] Jacob S (2013). Consumption pattern of nutritional health drinks and energy drinks among university students in Ajman, UAE.

[46] Harb JN, Taylor ZA, Khullar V, Sattari M: Rare cause of acute hepatitis: a common energy drink. BMJ Case Rep 2016, 2016. 10.1136/bcr-2016-216612 PMC5129143 27803015

[47] Bawazeer Naif A., AlSobahi Najmah A. (2013). Prevalence and Side Effects of Energy Drink Consumption among Medical Students at Umm Al-Qura University, Saudi Arabia. International Journal of Medical Students.

[48] Bassiony MM (2009). Smoking in Saudi Arabia. Saudi Med J.

[49] Hukkanen J, Jacob P, Peng M, Dempsey D, Benowitz NL (2011). Effect of nicotine on cytochrome P450 1A2 activity. Br J Clin Pharmacol.

[50] Miller KE (2008). Energy drinks, race, and problem behaviors among college students. J Adolesc Health.

[51] Alford C, Scholey A, Verster JC (2015). Energy drinks mixed with alcohol: are there any risks?. Nutr Rev.

[52] Berger LK, Fendrich M, Chen HY, Arria AM, Cisler RA (2011). Sociodemographic correlates of energy drink consumption with and without alcohol: results of a community survey. Addict Behav.

[53] Persad LA (2011). Energy drinks and the neurophysiological impact of caffeine. Front Neurosci.

