# Supplementary material for: Energy drinks and population health: consumption pattern and adverse effects among Saudi population
Source: BMC Public Health. 2019 Nov 21;19:1539. doi: 10.1186/s12889-019-7731-z (PMC6869250; doi:10.1186/s12889-019-7731-z)
Supplement: Supplementary file 1 — Additional file 1. Study Questionnaire: The English version of the questionnaire that was used to obtain the data from study participants. [file 12889_2019_7731_MOESM1_ESM.docx]

Energy Drinks and Population Health: Consumption Pattern and Adverse Effects among Saudi Population

The aim of this questionnaire is to study the opinion and attitude toward energy drinks and population health, which may help to find out the knowledge, consumption pattern and benefit as well as the adverse effect of energy drinks on human health in the kingdom of Saudi Arabia especially in the Hail region.

Please be advised that all information will be dealt with in strict confidence and will be used for research purposes only.

***Please tick* (√) *your response to the appropriate answer***

| 1. **Sociodemographic characteristics of study participants and select health habits** |
| --- |

**A.1. Gender:**

- Male
- Female

**A.2. Age group:**

- 15-18 years
- 19-25 years
- 26-40 years
- ≥41 years

**A.3. Nationality:**

- Saudi
- Non-Saudi

**A.4. Education:**

- No formal education
- Less than high school
- High school
- College student
- Bachelor degree
- Masters degree
- Ph.D degree

**A.5. Your sleeping habit:**

- Regular
- Irregular

**A.6. Sleep duration:**

- 5 hours or less
- 6-8 hours
- More than 8 hours

**A.7. Do you smoke?**

- Yes
- No

**A.8. Your self-health awareness:**

- Good Health
- Bad Health

| 1. **Knowledge about energy drinks among consumers** |
| --- |

**B.1. Do energy drinks raise blood pressure?**

- Yes
- No

**B.2. Do energy drinks affect glucose levels?**

- Yes
- No

**B.3.** **Do energy drinks affect heart rate?**

- Yes
- No

**B.4. Do energy drinks cause possible side effects?**

- Yes
- No

**B.5. Do you recommend energy drinks to others?**

- Yes
- No

**B.6. What can be a suitable replacement for energy drinks?**

- Coffee
- Tea
- Natural herbs
- Others:

**B.7. Do you think Saudi Food and Drug Authority (SFDA) should regulate consumption of energy drinks?**

- Yes
- No

| 1. **Consumption pattern of energy drinks** |
| --- |

**C.1. What is your average consumption of energy drinks?**

- Daily
- More than once weekly
- Once weekly
- 1-3 times monthly

**C.2. Why do you drink energy drinks?**

- To reduce fatigue
- To concentrate during studying
- To be more alert and awaken
- I like it
- Others:

**C.3. What is your energy drinks preferred consumption time?**

- In the morning
- With meals
- At night
- Anytime

**C.4. What activities are related to your energy drinks consumption?**

- Meetings and celebrations
- Study exams
- Others:

**C.5. What is your preferred energy drink brand?**

- Bison
- Red Bull
- Power Horse
- Bugzy
- Code Red
- Black
- Smart Cola
- Vitamin C

**C.6. What is your reason for selection of your preferred bran?**

- Taste
- Strong effect
- Price
- Other:

| 1. **Perceived desirable effects of energy drinks** |
| --- |

**D.1. Do you feel mood elevation when you consume energy drinks?**

- Yes
- No

**D.2. Do you become more energetic when you consume energy drinks?**

- Yes
- No

**D.3. Do you think energy drinks help in athletic and academic performance?**

- Yes
- No

**D.4. Do you think energy drinks help in concentration and memory recall?**

- Yes
- No

**D.5. Do energy drinks help you in driving long trips?**

- Yes
- No

| 1. **Experienced adverse events associated with energy drinks consumption** |
| --- |

**E.1. Have you experienced tremors?**

- Yes
- No

**E.2 Have you experienced chest pain?**

- Yes
- No

**E.3.** **Have you experienced palpitations?**

- Yes
- No

**E.4. Have you suffered from insomnia?**

- Yes
- No

**E.5. Have you experienced headaches?**

- Yes
- No

**E.6. Have you experienced constipation?**

- Yes
- No

**E.7. Have you experienced diuresis?**

- Yes
- No

**E.8.** **Have you experienced chronic fatigue?**

- Yes
- No

**E.9. Have you experienced muscle fatigue?**

- Yes
- No

**E.10. Have you experienced confusion?**

- Yes
- No

**E.11. Have you suffered from tooth decay?**

- Yes
- No

**E.12. Have you felt lack of rest?**

- Yes
- No

**E.13.** **Have you experienced nervousness?**

- Yes
- No

**E.14. Have you experienced Nausea/Abdominal pain?**

- Yes
- No

**E.15. Have you suffered from any other adverse event?**

- Yes:
- No

**E.16. Have you tried to quit consuming energy drinks?**

- Yes
- No

**E.17. If you used to drink energy drinks a lot then you quit for a while, did you suffer from any withdrawal symptoms?**

- Yes:
- No
